# Analysis of the Sex-Specific Risk Factors for Arterial Stiffness

**DOI:** 10.31083/RCM25478

**Published:** 2025-02-20

**Authors:** Jinhuan Yuan, Chengwen Wang, Chong Zhao, Hua Liu, Yiwen Zhang, Meitong Liu, Tianxiang Fu, Shouling Wu

**Affiliations:** ^1^Zhifu Center for Disease Control and Prevention, 264000 Yantai, Shandong, China; ^2^Yantai Engineering & Technology College, 264000 Yantai, Shandong, China; ^3^Department of Cardiology, Kailuan Hospital, Hebei United University, 063000 Tangshan, Hebei, China

**Keywords:** sex-specific, arteriosclerosis, baPWV, risk factors

## Abstract

**Background:**

To explore the sex-specific risk factors of associated with arterial stiffness.

**Methods:**

A total of 28,291 participants from the Kailuan study cohort were enrolled in this study. A multivariate linear regression analysis and a multivariate logistic regression model were used to analyze the influencing factors of arteriosclerosis (indexed using the brachial–ankle pulse wave velocity, baPWV) between different sexes.

**Results:**

The incidence of arteriosclerosis (baPWV greater than or equal to 1400 cm/s) was 54.70%. The incidence of arteriosclerosis in males (62.13%) was higher than in females (37.41%) (*p* < 0.01). According to age stratification (5 years difference for each group), the baPWV values of males in all age groups <70 years were higher than in females (*p* < 0.01). The increase in baPWV values was higher in females over 45 years than in males and correlated with males in the 70–75 age group. The multivariate linear regression model showed that for every 5-year increase in age, the baPWV increased by 62.55 cm/s in males and 71.86 cm/s in females. Furthermore, for every 10 mmHg increase in systolic blood pressure (SBP), the baPWV increased by 61.01 cm/s in males and 51.86 cm/s in females. Regular physical exercise reduced the baPWV in males, but there was no statistical correlation in females. The waist-to-hip ratio (WHR) increased the baPWV in females yet was not statistically significant in males. Multivariate logistic regression analysis showed that after adjusting for confounding factors (age, WHR, SBP, heart rate, triglyceride, low-density lipoprotein cholesterol (LDL-C), high-density lipoprotein cholesterol (HDL-C), high-sensitivity C-reactive protein (hs-CRP), estimated glomerular filtration rate (eGFR), diabetes, higher education, higher income, smoking, drinking, and physical exercise), males were 1.89 times more likely than females to develop arteriosclerosis (*p* < 0.05). A stratified analysis of males and females showed that the risk of arteriosclerosis was higher in females than in males in the 45–60 and over 60 age groups compared with those in the under 44 age group (*p* < 0.01). Diabetes, LDL-C, and hs-CRP were more likely to be correlated with arteriosclerosis in females than in males (odds ratio (OR): 2.32, 1.26, 1.08 *vs*. 1.83, 1.17, 1.02, respectively, *p* < 0.05). Higher education levels reduced the risk of arteriosclerosis in males and females, with OR values of 0.64 and 0.84, respectively (*p* < 0.05).

**Conclusions:**

The arteriosclerosis detection rate in males was higher than in females. Conversely, the increase in baPWV in females older than 45 years was higher than in males. Meanwhile, WHR, diabetes, LDL-C, and hs-CRP were more likely to be correlated with arteriosclerosis in females.

**Clinical Trial Registration:**

Chinese Clinical Trail Registry, URL: https://www.chictr.org.cn/showproj.html?proj=8050. Unique identifier: ChiCTR-TNRC-11001489 .

## 1. Introduction

Arteriosclerosis is a process whereby the arterial wall thickens, resulting in decreased wall compliance, loss of elasticity, and narrowing of the lumen [1]. Increased arterial stiffness increases the risk of hypertension [2], coronary heart disease [3], stroke [4], renal dysfunction [5], cognitive dysfunction [6], and peripheral vascular disease [7]. Therefore, early prevention of increased arterial stiffness significantly reduces the risk of cardiovascular and cerebrovascular diseases. However, the compliance of the aorta decreases, and the stiffness increases with age [8]. The Framingham study found that females, hypertension, increased body mass index, and diabetes were also risk factors for arteriosclerosis [9]. Other studies found that uric acid excretion fraction and estimated glomerular filtration rate were inversely proportional to the brachial–ankle pulse wave velocity (baPWV) [10, 11]. However, regular aerobic exercise can reduce the degree of arteriosclerosis [12].

Carotid–femoral pulse wave velocity (cfPWV) is the gold standard for predicting arteriosclerosis [7, 13], yet measuring the cfPWV is relatively complex. Notably, the baPWV and cfPWV exhibit a good correlation. The baPWV method is simple and reproducible and is a sensitive index to evaluate arteriosclerosis [14]. The guidelines and consensus of China on the Prevention and Treatment of Cardiovascular Disease published in 2008 consider a baPWV ≥1400 cm/s to be consistent with arteriosclerosis [15].

The baPWV differs among different sex groups. Wang X* et al*. [16] found that in patients under 50 years, the baPWV was higher in males than in females, and when the age was over 50, there was no statistical difference between baPWV in males *vs*. females. Benetos *et al*. [17] found the same phenomenon in patients ≤80 years old and >80 years old. Some studies have suggested the possibility that these differences are due to age [16, 17, 18], height [19], blood pressure [20], and sex hormones [21]. However, no studies exist on the differences in the factors affecting arterial stiffness among different sexes in China. Therefore, we analyzed those factors responsible for arterial stiffness in Chinese males and females.

## 2. Materials and Methods

### 2.1 Study Participants

Data were derived from the Kailuan study. Briefly, the Kailuan study is a prospective, community-based cohort study that aimed to investigate the epidemiology of cardiovascular diseases in Chinese adults. All participants underwent assessments via questionnaires, clinical examinations, and laboratory tests upon enrollment and were followed up every two years. Details regarding data collection were described previously [22, 23, 24, 25, 26].

The study enrolled participants older than 18 years who underwent baPWV measurements from 2010 to 2017. Exclusion criteria included a history of myocardial infarction, stroke, cancer, or peripheral vascular disease before the baPWV measurement. The ethics committees at Kailuan General Hospital approved the study, following the guidelines outlined by the Helsinki Declaration. Written informed consent was obtained from all participants. Baseline data on demographics and cardiovascular risk factors were collected when the baPWV measurements were performed.

### 2.2 Measurements for baPWV

We collected baPWV values using a BP-203 RPE III networked arteriosclerosis detection device produced by Omron Health Medical (Liaoning, China) Co., Ltd. Participants underwent baPWV measurements after at least 5 minutes of rest in the supine position. Cuffs were wrapped on both arms and ankles. The lower edge of the arm cuff was positioned 2–3 cm above the cubital fossa transverse striation, while the lower edge of the ankle cuff was positioned 1–2 cm above the medial malleolus. The heartbeat monitor was placed on the left edge of the sternum, and electrocardiogram electrodes were placed on both wrists. The resulting baPWV value could be directly read using the network connection. The methodology for the baPWV measurement remained constant for all participants.

### 2.3 Definition of Arteriosclerosis

The definition for arteriosclerosis was derived from the American Heart Association Medical/Scientific Report (1993) criteria: baPWV <1400 cm/s for normal arterial stiffness, baPWV ≥1400 cm/s for arteriosclerosis. Likewise, the guideline and Consensus 2008 for Cardiovascular Disease Prevention and Treatment defines a baPWV ≥1400 cm/s as arteriosclerosis [15].

### 2.4 Statistical Analysis

We presented continuous variables as the mean ± standard deviation (SD) or median with interquartile range and categorical variables as percentages. The baseline characteristics of participants were compared between females and males using *t*- or Wilcoxon tests for continuous variables and χ^2^ test for categorical variables.

We examined cross-sectional correlates of arteriosclerosis (baPWV ≥1400 cm/s) using multiple linear regression analysis and multivariable backward logistic regression in males and females, retaining covariates with a *p*-value < 0.05, including age, sex, body mass index (BMI), alcohol consumption, physical exercise, history of diabetes, history of hyperlipidemia, education level, fasting blood glucose (FBG), total cholesterol, low-density lipoprotein cholesterol (LDL-C), high-sensitivity C-reactive protein (hs-CRP), and use of antidiabetic and lipid-lowering drugs.

Two-sided *p*-values < 0.05 were considered statistically significant. All analyses were performed using SAS version 9.4 (SAS Institute Inc., Cary, NC, USA).

## 3. Results

### 3.1 Study Participants

**Fig. 1.**
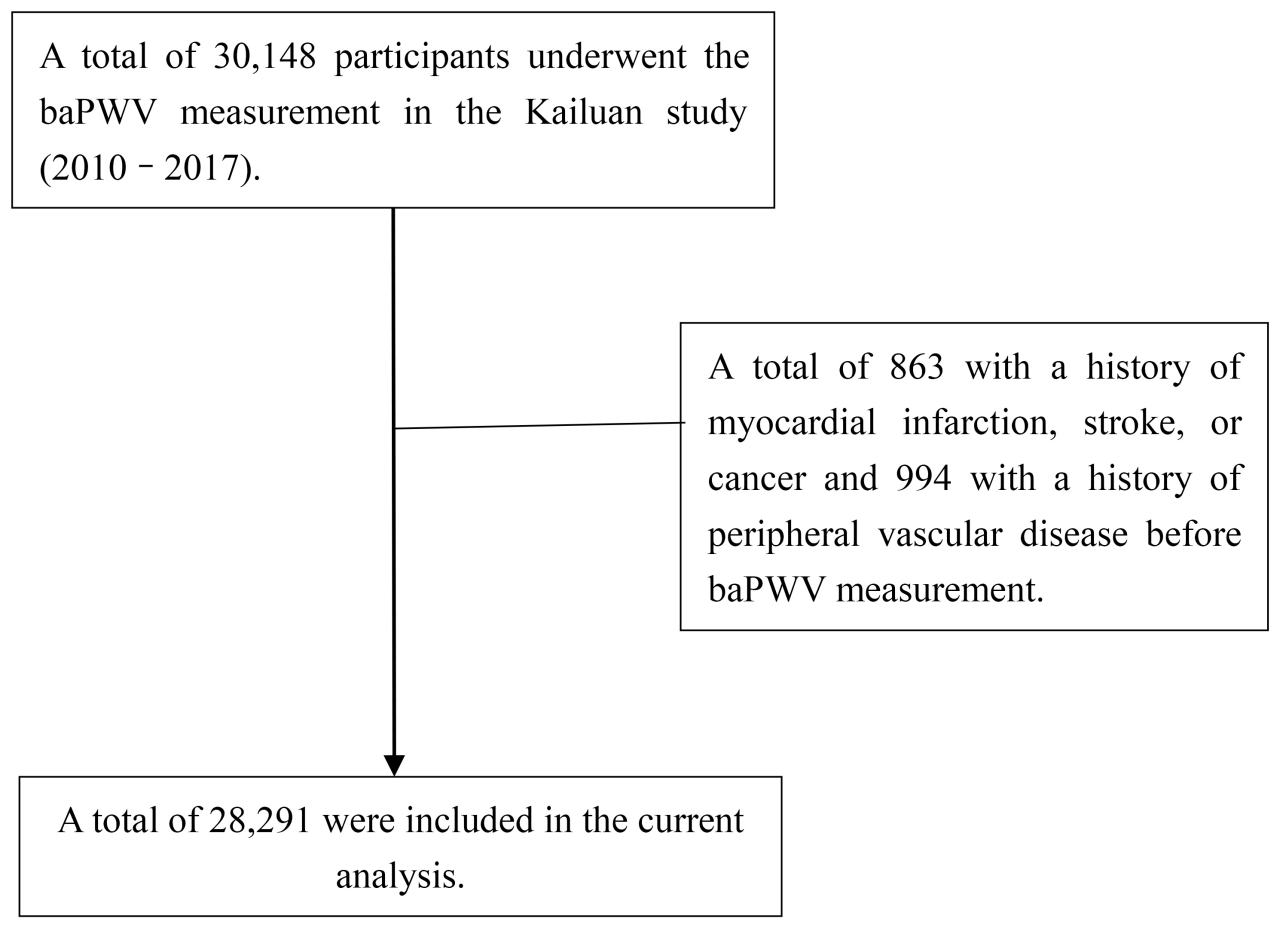
**Flow diagram of the patient selection in the current analysis**. baPWV, brachial–ankle pulse wave velocity.

From January 1, 2010, to December 31, 2017, 30,148 participants in the Kailuan study underwent baPWV measurements. After excluding participants with a history of myocardial infarction, stroke, cancer, or peripheral vascular disease, 28,291 participants were included in the current analysis (Fig. 1).

### 3.2 Comparison of Baseline Characteristics

The average age among the 28,291 participants was 48.12 ± 12.53 years, and 19,783 were males (69.93%). Compared with females, males had a higher baPWV, mean age, BMI, blood pressure (systolic blood pressure (SBP), diastolic blood pressure (DBP)), heart rate, estimated glomerular filtration rate (eGFR), hypertension, diabetes, hyperlipidemia, smoking, and alcohol consumption (*p* < 0.01). Females administered more lipid-lowering drugs and had higher education levels than males (*p* < 0.01) (Table 1).

**Table 1.** **Comparison of baseline characteristics in the different sex groups**.

Variable	Males (n = 19,783)	Females (n = 8508)	*t*/χ^2^	*p*
Age (y)	48.49 ± 12.84	47.25 ± 11.73	7.94	<0.01
baPWV (cm/s)	1550.37 ± 336.16	1387.85 ± 327.96	37.90	<0.01
BMI (kg/m^2^)	25.18 ± 3.25	24.26 ± 3.63	19.61	<0.01
WHR	0.92 ± 0.07	0.87 ± 0.10	35.16	<0.01
SBP (mmHg)	133.06 ± 17.28	122.25 ± 19.07	43.91	<0.01
DBP (mmHg)	83.84 ± 10.71	77.90 ± 10.15	43.44	<0.01
Uric acid (mmol/L)	330.42 ± 99.57	262.43 ± 69.81	64.32	<0.01
Heart rate (bpm)	74.55 ± 11.42	72.99 ± 9.59	10.46	0.01
FBG (mmol/L)	5.85 ± 2.18	5.46 ± 1.66	16.05	<0.01
Triglyceride (mmol/L)	1.86 ± 2.50	1.42 ± 2.22	14.29	<0.01
LDL-C (mmol/L)	2.77 ± 1.15	2.56 ± 0.93	16.45	<0.01
HDL-C (mmol/L)	1.43 ± 0.82	1.53 ± 0.51	–11.44	<0.01
hs-CRP (mg/L)	2.01 ± 4.52	1.88 ± 3.08	2.61	<0.01
eGFR (mL/min/1.73 m^2^)	103.13 ± 27.68	98.43 ± 21.62	15.22	<0.01
Hypertension	8451 (42.66)	2140 (25.23)	770.58	<0.01
Diabetes	2834 (14.31)	795 (9.37)	129.29	<0.01
Dyslipidemia	12,363 (62.41)	4281 (50.47)	349.55	<0.01
Antihypertensive	2765 (15.20)	1034 (12.56)	32.00	<0.01
Antidiabetic	906 (4.97)	386 (4.69)	1.00	0.32
Lipid-lowering	174 (1.31)	85 (1.61)	2.38	0.12
High school or above	4330 (32.73)	3297 (50.46)	579.84	<0.01
Monthly income USD ≥800	1012 (7.90)	421 (6.91)	5.78	0.02
Current smoker	9542 (50.72)	146 (1.85)	5730.73	<0.01
Current alcohol drinker	1664 (9.17)	16 (0.19)	767.38	<0.01
Physical exercise	2164 (11.86)	1010 (12.23)	0.75	0.39

Continuous variables are presented as the mean ± SD or median with interquartile range and categorical variables as percentages. baPWV, brachial–ankle pulse wave velocity; BMI, body mass index; WHR, waist-to-hip ratio; SBP, systolic blood pressure; DBP, diastolic blood pressure; FBG, fasting blood glucose; LDL-C, low-density lipoprotein cholesterol; HDL-C, high-density lipoprotein cholesterol; hs-CRP, high-sensitivity C-reactive protein; eGFR, estimated glomerular filtration rate.

### 3.3 Prevalence of Arterial Stiffness

**Fig. 2.**
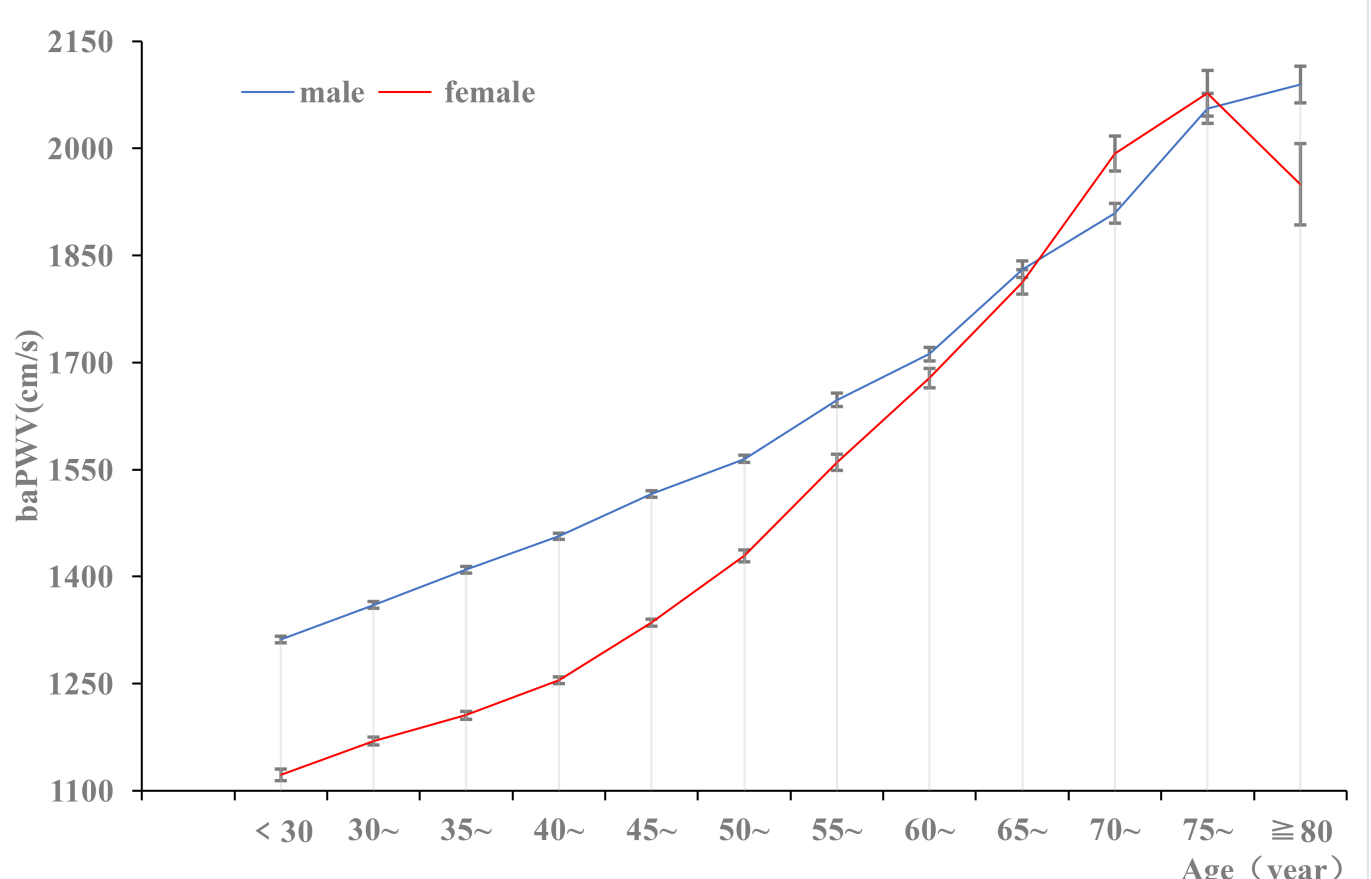
**The prevalence of baPWV in different sex groups**. baPWV, brachial–ankle pulse wave velocity.

The detection rate of arteriosclerosis in males (62.13%) was higher than in females (37.41%) (*p* < 0.01). The changes in baPWV were described by age stratification (over 30 years old according to an increase in age of 5 years). The baPWV increased with age in the different sex groups (*p* < 0.05) (Table 2). The baPWV in males was higher than in females in all age groups <70 years old (*p* < 0.01). However, from age 45 years and older, the baPWV increased faster in females than in males (Fig. 2).

**Table 2.** **Distribution of baPWV in different sex groups**.

Age (y)	Males	Ratio (%)	Females	Ratio (%)	*p*
baPWV ≥1400 (cm/s) (N(%))	12,292 (62.13)	69.93	3183 (37.41)	30.07	<0.01
<30	1310.24 ± 192.85	8.71	1120.81 ± 159.44	4.68	<0.01
30–35	1361.09 ± 183.94	7.58	1168.13 ± 166.08	10.41	<0.01
35–40	1408.89 ± 200.18	9.14	1202.75 ± 168.65	10.28	<0.01
40–45	1454.68 ± 227.99	13.78	1254.82 ± 179.64	17.06	<0.01
45–50	1518.98 ± 273.38	17.40	1335.88 ± 216.93	22.54	<0.01
50–55	1570.81 ± 303.89	17.20	1430.11 ± 243.66	9.83	<0.01
55–60	1667.35 ± 361.34	7.07	1563.88 ± 306.22	8.49	<0.01
60–65	1738.61 ± 374.81	6.88	1684.94 ± 352.76	7.65	<0.01
65–70	1839.59 ± 376.42	4.96	1818.30 ± 332.09	4.23	0.50
70–75	1918.36 ± 375.16	3.42	1997.92 ± 387.15	2.57	0.05
75–80	2077.59 ± 440.69	1.95	2108.83 ± 419.63	1.532	0.66
≥80	2120.16 ± 538.64	1.91	1992.39 ± 501.22	0.72	0.62
*p*-trend	*p * < 0.05	–	*p * < 0.05	–	–

baPWV, brachial–ankle pulse wave velocity.

### 3.4 Determinants of Arterial Stiffness in Different Sex Groups

Using baPWV as the dependent variable, the statistically significant correlated factors (Table 3) were entered into the multiple linear regression model; eight statistically significant factors were identified in the male and female populations. For every increase of 5 years in age, the baPWV in males increased by 62.55 cm/s and 71.86 cm/s in females. For every 10 mmHg increase in the SBP, the baPWV increased in males and females by 61.01 cm/s and 51.86 cm/s. Physical exercise significantly reduced the baPWV in males but was not statistically significant in females. Females with a higher WHR demonstrated a greater, although not significantly different, baPWV than males (Table 4).

**Table 3.** **The relationship between various influencing factors and baPWV**.

Variable	Total		Males		Females	
	r	*p*	r	*p*	r	*p*
Sex	–0.223	<0.01	-	-	-	-
Age (y)	0.575	<0.01	0.520	<0.01	0.686	<0.01
WHR	0.142	<0.01	0.049	<0.01	0.142	<0.01
SBP (+10 mmHg)	0.546	<0.01	0.476	<0.01	0.599	<0.01
Heart rate (bpm)	0.151	<0.01	0.144	<0.01	0.119	<0.01
Triglyceride (mmol/L)	0.088	<0.01	0.049	<0.01	0.122	<0.01
LDL-C (mmol/L)	0.084	<0.01	0.015	0.15	0.165	<0.01
HDL-C (mmol/L)	0.001	0.94	0.042	<0.01	–0.029	0.047
hs-CRP (mg/L)	0.08	<0.01	0.067	<0.01	0.123	<0.01
Uric acid (mmol/L)	0.122	<0.01	0.002	0.86	0.190	<0.01
eGFR (mL/min/1.73 m^2^)	–0.160	<0.01	–0.186	<0.01	–0.21	<0.01
Diabetes	0.257	<0.01	0.227	<0.01	0.305	<0.01
High school or above	–0.208	<0.01	–0.137	<0.01	–0.258	<0.01
Monthly income USD ≥800	–0.018	0.03	–0.029	0.004	–0.003	0.86
Salt bias	0.017	0.04	–0.012	0.25	0.025	0.08
Current smoker	0.065	<0.01	–0.066	<0.01	0.018	0.21
Current alcohol drinker	0.057	<0.01	0.02	0.047	0.01	0.51
Physical exercise	0.105	<0.01	0.096	<0.01	0.143	<0.01

baPWV, brachial–ankle pulse wave velocity; WHR, waist-to-hip ratio; SBP, systolic blood pressure; LDL-C, low-density lipoprotein cholesterol; HDL-C, high-density lipoprotein cholesterol; hs-CRP, high-sensitivity C-reactive protein; eGFR, estimated glomerular filtration rate.

**Table 4.** **Multiple linear regression analysis of baPWV-related factors in different sex groups**.

Sex	Variable	β	SE	SD	t	*p*
Males	Age (+5 y)	62.55	1.58	0.41	39.54	<0.01
	SBP (+10 mmHg)	61.01	2.08	0.31	29.36	<0.01
	Heart rate (bpm)	4.81	0.32	0.15	15.11	<0.01
	Physical exercise	–25.21	9.37	–0.03	–2.69	0.01
	Dyslipidemia	15.40	7.2	0.02	2.14	0.03
	Diabetes	71.48	9.75	0.07	7.33	<0.01
	Triglyceride (mmol/L)	3.43	1.41	0.02	2.43	0.02
Females	Age (+5 y)	71.86	2.29	0.46	31.41	<0.01
	SBP (+10 mmHg)	51.86	2.85	0.29	18.22	<0.01
	Heart rate (bpm)	4.75	0.49	0.12	9.75	<0.01
	WHR	179.45	68.43	0.03	2.62	<0.01
	Diabetes	67.63	14.87	0.06	4.55	<0.01
	HDL-C (mmol/L)	–27.13	10.19	–0.03	–2.66	<0.01

β, beta coefficient; SE, standard error; SD, standard deviation; baPWV, brachial–ankle pulse wave velocity; SBP, systolic blood pressure; WHR, waist-to-hip ratio; HDL-C, high-density lipoprotein cholesterol.

Using a multivariable logistic regression analysis, we found that the risk of arteriosclerosis in males was 1.89 times higher than in females (*p* < 0.01) (Table 5). The stratified analysis of males and females showed that the risk of arteriosclerosis was higher in females aged 45–60 years and older than those under 44 years and was higher than in males (*p* < 0.01). The correlation between diabetes and arteriosclerosis in females was stronger than in males (odds ratio (OR): 2.32 *vs*. 1.82, *p* < 0.01). Higher education levels reduced the risk of arteriosclerosis in males and females (OR: 0.64, 95% confidence interval (CI): 0.51–0.79; OR: 0.83, 95% CI: 0.74–0.95, respectively) (Table 6).

**Table 5.** **Multivariate logistic regression analysis of baPWV in males and females**.

Variable	β	Waldχ^2^	OR	95% CI	*p*
Sex (males)	0.61	146.02	1.89	1.73–2.06	<0.01
Age (45–59 y)	0.76	297.47	2.11	1.93–2.30	<0.01
Age (≥60 y)	2.38	991.78	10.63	9.11–12.40	<0.01
WHR	0.01	0.10	1.01	0.93–1.11	0.75
SBP (+10 mmHg)	0.55	1353.32	1.74	1.69–1.79	<0.01
Heart rate (bpm)	0.01	73.45	1.01	1.01–1.02	<0.01
Triglyceride (mmol/L)	0.06	23.00	1.06	1.04–1.09	<0.01
LDL-C (mmol/L)	0.13	26.09	1.13	1.08–1.19	<0.01
HDL-C (mmol/L)	–0.04	0.66	0.97	0.89–1.05	0.42
hs-CRP (mg/L)	0.02	8.33	1.02	1.01–1.03	<0.01
eGFR (mL/min/1.73 m^2^)	–0.09	0.87	0.91	0.75–1.11	0.35
Diabetes	0.84	116.03	2.26	1.93–2.65	<0.01
High school or above	–0.30	49.74	0.74	0.68–0.80	<0.01
Monthly income USD ≥800	–0.01	0.02	0.99	0.86–1.14	0.88
Salt bias	0.03	0.23	1.03	0.91–1.17	0.64
Current smoker	0.07	1.78	1.07	0.97–1.17	0.18
Current alcohol drinker	0.05	0.34	1.05	0.89–1.23	0.56
Physical exercise	0.08	1.76	1.11	0.98–1.26	0.09

β, beta coefficient; Waldχ^2^, Wald Chi-squared test; OR, odds ratio; CI, confidence interval; baPWV, brachial–ankle pulse wave velocity; WHR, waist-to-hip ratio; SBP, systolic blood pressure; LDL-C, low-density lipoprotein cholesterol; HDL-C, high-density lipoprotein cholesterol; hs-CRP, high-sensitivity C-reactive protein; eGFR, estimated glomerular filtration rate.

**Table 6.** **Multivariate logistic regression analysis of baPWV in different sex groups**.

Variable	Sex	β	Waldχ^2^	OR	95% CI	*p*
Males	-	0.50	68.97	1.65	1.46–1.85	<0.01
Age* (y)						
45–59	Males	0.59	71.8	1.80	1.57–2.07	<0.01
	Females	1.25	93.64	3.49	2.71–4.49	<0.01
≥60	Males	2.38	378.42	10.81	8.50–13.74	<0.01
	Females	3.19	293.26	24.19	16.80–34.83	<0.01
SBP (mmHg)	Males	0.49	442.47	1.63	1.56–1.71	<0.01
	Females	0.51	180.21	1.66	1.54–1.80	<0.01
Heart rate (bpm)	Males	0.03	73.32	1.03	1.02–1.03	<0.01
	Females	0.03	21.25	1.03	1.02–1.04	<0.01
Triglyceride (mmol/L)	Males	0.04	5.19	1.04	1.01–1.08	0.02
	Females	0.00	0.07	1.01	0.97–1.01	0.80
LDL-C (mmol/L)	Males	0.15	13.56	1.16	1.07–1.26	<0.01
	Females	0.24	11.03	1.27	1.10–1.46	<0.01
HDL-C* (mmol/L)	Males	–0.01	0.01	0.99	0.89–1.11	0.91
	Females	–0.31	6.69	0.73	0.58–0.93	0.01
hs-CRP (mg/L)	Males	0.02	5.47	1.02	1.01–1.04	0.02
	Females	0.08	14.59	1.08	1.04–1.12	<0.01
Diabetes*	Males	0.60	22.76	1.82	1.43–2.34	<0.01
	Females	0.84	17.64	2.32	1.57–3.43	<0.01
Education*	Males	–0.18	7.92	0.83	0.74–0.95	<0.01
	Females	–0.46	17.07	0.64	0.51–0.79	<0.01

* indicates variable by sex in interaction. β, beta coefficient; Waldχ^2^, Wald Chi-squared test; OR, odds ratio; CI, confidence interval; baPWV, brachial–ankle pulse wave velocity; SBP, systolic blood pressure; LDL-C, low-density lipoprotein cholesterol; HDL-C, high-density lipoprotein cholesterol; hs-CRP, high-sensitivity C-reactive protein.

## 4. Discussion

Our study found that the prevalence of arteriosclerosis in males was higher than in females and that the baPWV in both males and females showed a trend of increasing with age. In all age groups <65 years old, the baPWV values in males were higher than in females. However, starting at 45 years, the baPWV values for females increased with age and were higher than for males over 71. Vermeersch *et al*. [27] found that in individuals over 45 years, the baPWV increased more rapidly in females than in males. Benetos *et al*. [17] found that the baPWV values in males were higher than in females before age 60. This indicates that arteriosclerosis exists in the process of physiological senescence, and the incidence of arteriosclerosis in females correlates more with aging. A possible explanation for the acceleration in baPWV increase in women over 45 years is that estrogen levels decrease, and arterial stiffness increases significantly in menopausal women [28]. Studies have also reported that androgen increased arterial stiffness by altering the arterial structures [21, 29], which may also be responsible for the baPWV values in males and females before age 65.

In addition to age factors, this study found that SBP, heart rate, uric acid, diabetes mellitus, triglyceride, and LDL-C levels were positively correlated with the baPWV in males and females, and for every 10 mmHg increase in SBP, the baPWV in males and females increased by 61.01 cm/s and 51.86 cm/s, respectively. The BLSA study found that elevated SBP was significantly associated with elevated baPWV and exhibited a stronger correlation in the male population [20]. Physical exercise reduced baPWV values in males, which was not statistically different from females, while high-density lipoprotein cholesterol (HDL-C) was the opposite. Heart rate and diabetes promoted similar changes in male and female baPWV values.

After adjusting for confounding factors, the risk of arteriosclerosis in males was 1.89 times higher than in females (95% CI: 1.73–2.06). Moreover, the risk of arteriosclerosis increased by 9.63 times in the group aged over 60 years. The risk of arteriosclerosis increases by 0.74 times for every 10 mmHg increase in SBP. The risk of arteriosclerosis in the diabetic population is 2.26 times greater than in the non-diabetic population. In addition, heart rate, triglyceride, and LDL-C are also risk factors for arteriosclerosis. This shows that being male is an independent risk factor for arteriosclerosis, and age is the most significant factor in increased arterial stiffness. Blood pressure, diabetes, heart rate, and blood lipid levels also increase arterial stiffness to varying degrees. Conversely, having a higher education was a protective factor against arteriosclerosis (OR: 0.74, 95% CI: 0.68–0.80).

By analyzing the influencing factors of arterial stiffness by sex, it was found that age, SBP, heart rate, LDL-C, hs-CRP, and diabetes are the risk factors for increased baPWV values in males and females.Compared with people aged ≤44, the risk of arteriosclerosis in females aged 45–59 and ≥60 is 3.49 times and 24.19 times, that in males aged 1.80 times and 10.81 times, indicating that the correlation between age and female arterial stiffness is higher than in male. Compared with the non-diabetic population, the risk of arteriosclerosis in female diabetic patients is higher than that in male (OR: 2.32–1.82). Higher education levels reduced the risk of arteriosclerosis in males and females (OR: 0.83, 95% CI: 0.74–0.95 *vs*. OR: 0.64, 95% CI: 0.51–0.79, respectively). Comparatively, individuals with higher education levels may have a healthier lifestyle; thus, a variable lifestyle is the main factor influencing the increase in arteriosclerosis, which may correlate to why a higher education level reduces the risk of arteriosclerosis. The proportion of females with higher education (50.46%) is higher than that of males (32.73%), which may be why the OR value for females is lower than for males. HDL-C reduces the risk of arteriosclerosis in females (OR: 0.73, 95% CI: 0.58–0.93), and there is no statistical difference between HDL-C and males. Lebrun *et al*. [30] also found that increased HDL-C reduced arterial stiffness. SBP, heart rate, LDL-C, and hs-CRP have little difference in increasing the risk of arteriosclerosis in males and females, and the OR values are 1.63, 1.03, 1.16, 1.02 and 1.66, 1.03, 1.27, and 1.08, respectively.

Our study found significant differences in arterial stiffness between different sexes. Age, blood pressure, diabetes, LDL-C, heart rate, and hs-CRP are arteriosclerosis risk factors. Age and diabetes are more related to arterial stiffness in females than males, and blood pressure pertains more to arterial stiffness in males than females. Similarly, aging and blood pressure also increase the risk of arteriosclerosis in males and females, respectively. Therefore, actively monitoring and controlling blood pressure, blood sugar, and blood lipids to maintain them at relatively normal and stable levels can reduce the occurrence and development of subclinical arteriosclerosis.

Although our study found that the influencing factors of arterial stiffness are different between sexes, this study has some limitations: (1) cfPWV, the gold standard of predicting arteriosclerosis, was not used as the detection method. However, baPWV not only presents a good correlation with cfPWV but was also included in the recommended standard of arteriosclerosis evaluation by the American Heart Association [7]. (2) There is a lack of data on whether the female subjects were or were not menopausal, and the reasons for the accelerated growth of baPWV after age 45 are uncertain. (3) Furthermore, this study did not exclude the influence of patients taking antihypertensive, hypoglycemic, and lipid-lowering drugs from the results, meaning that it cannot be ruled out that such people were not included in the sensitivity analysis. (4) When analyzing the differences in influencing factors of arterial stiffness between different sexes, although possible confounding factors were corrected for as much as possible, other confounding factors exist, such as environmental changes and heritage factors, which were not corrected.

## 5. Conclusions

The detection rate of arteriosclerosis in males was higher than in females. The baPWV value in males under 70 years was higher than in females. However, after 45 years, the increase in the baPWV rate in females according to age was higher than in males. The WHR, SBP, diabetes, LDL-C, and hs-CRP measurements correlated more with female arteriosclerosis. Higher education and physical exercise levels reduced the risk of arteriosclerosis in males and females.

## Availability of Data and Materials

The data are obtainable on request from the corresponding author in this study. They are not publicly available due to privacy issues.
